# Chicago Pathological Society

**Published:** 1882-11

**Authors:** 


### ARTICLE IX.

THE CHICAGO PATHOLOGICAL SOCIETY. Stated Meeting, October 9, 1882. Dr. H. M. Lyman, President in the chair.

### THE TUBERCLE BACILLUS.

Dr. W. T. Belfield extemporized a lecture on the above subject, and said that the whole matter of bacteria had of late fallen in disrepute and ridicule in the opinion of the profession. There had been so many hasty conclusions reached that the profession might indeed be sceptical. A little original work would convince one, however, that there was a great deal of fire underneath the smoke. There were as yet two well known diseases whose immediate cause had been proved to be a parasitic micro-organism. The first was anthrax, also called charbon, and splenic fever. It had been clearly proved that the disease was immediately dependent on a bacillus. The second disease was not known in this country. It was first discovered in 1877, and is a primary disease of cattle *actino-mycosis bovis*, though we are just as liable to catch it when exposed. Although recognized only five years ago, the parasites are so large as to be easily investigated. The disease consists in a series of tumors about the jaws which give rise to subacute abscesses; it is self limited in duration and results in recovery, in cattle, but in man it is by no means amenable to treatment. This is perhaps on account of the erect posture of man, for the abscesses give rise in him to pleuritis and peritonitis, reaching to the peritoneum from the mediastinum. Of eighteen cases now on record, only a few recovered. It is by no means determined just in what manner the bacillus causes the disease. But when the parasites are cultivated in gelatine with sufficient care, and then inoculated through an abrasion of the skin or mucous membrane into an animal, other viruses being excluded, the parasites appear in the blood within thirty hours. They are seen first in the blood vessels leading from the seat of inoculation, but in forty-eight hours the blood is full of bacilli through the body. Death generally takes place in three days and, at the autopsy, the capillaries of the various organs are found clogged with parasites. It seems that the circulation of the blood would be rendered impossible under such circumstances. Yet, whether a mechanical obstruction of the blood, or a noxious change in this fluid causes death is not certain, but what is well known is that the bacilli *are* the cause of death. (Here the bacillus anthracis was exhibited to all the members present, and these organisms were very distinct under even a low magnifying power).

As to the *tubercular bacillus*, said Dr. Belfield, there seems to be widely different opinions entertained as to the value of its presence in the tubercles. Their causative relations rested on the experiments of Koch, who had given the subject much attention for the last two years, and he was certainly the most experienced, accurate and trustworthy investigator of micro-organisms in our day. Medical men as yet had exhibited much reserve in receiving his statements regarding these bacilli. The connection of Koch with the Board of Health of Berlin, had given him all the advantages required for his researches.

Reports of many clinical observations of the bacilli have been made. Professional pathologists have been devoting time to the study of the presence of the bacilli in the expectorations of phthisical patients; and the reports are unanimous in representing the bacilli in the sputa examined. So that there is at least good presumptive evidence that Koch's views are correct. Such was the stand of the subject in Europe to-day.

In a long category of diseases, all septic diseases, bacteria have been repeatedly found, but no experimental proofs of causal relations have been furnished, except in the three diseases just spoken of. Dr. Belfield had no doubt but that the same convincing proofs would in time be furnished for every one of them. While the term, bacteria, was applied to all micro-organisms, a bacillus was a rod-shaped bacterium, while a micrococcus was a spherical one. Only bacilli, so far, were known to cause diseases, while micrococci were found in all decomposing organic matter, and were always found in the infectious diseases, as diphtheria. The organism of actino-mycosis is not a bacterium, but resembles an alga. It was a question whether the examination of sputa in phthisis had any clinical value. Dr. Belfield had examined twenty-two specimens in an hospital. In all of them the bacilli were found. Upon inquiring of the pathologist who furnished them, it was found that twenty were cases of well marked phthisis, while the two remaining cases were inscribed as chronic bronchitis. But, as the doctor remarked, the demarcation line between the two diseases is often obscure.

Here Dr. Belfield exhibited the bacilli magnified 250, nd every member looked at them. It used to require a magnifying power of 800 to 1000, in order to see the tubercle bacilli, but his mode of preparing them did not necessitate such objectives. The bacillus is $\frac{1}{6000}$ x $\frac{1}{20}$ inch in size.

At the same meeting, Dr. J. H. Tebbetts exhibited a specimen of cancer of the stomach, and this was followed by a voluminous paper on sleep, by Prof. Lyman. It is a contribution to the *International Cyclopœdia of Surgery*, and is, no doubt, the most valuable article ever written on the subject of sleep and its allied states. Somnambulism, hypnotism, and trance receive a careful treatment and their various causes were explained.

